# Intranasal drug delivery of small interfering RNA targeting Beclin1 encapsulated with polyethylenimine (PEI) in mouse brain to achieve HIV attenuation

**DOI:** 10.1038/s41598-017-01819-9

**Published:** 2017-05-12

**Authors:** Myosotys Rodriguez, Jessica Lapierre, Chet Raj Ojha, Ajeet Kaushik, Elena Batrakova, Fatah Kashanchi, Seth M. Dever, Madhavan Nair, Nazira El-Hage

**Affiliations:** 1 Department of Immunology, Florida International University, Herbert Wertheim College of Medicine, Miami, FL 33199 USA; 2 University of North Carolina, Eshelman School of Pharmacy, Chapel Hill, NC 27599 USA; 3 Laboratory of Molecular Virology, School of Systems Biology, George Mason University, Manassas, VA 20110 USA

## Abstract

We previously reported that activation of the host autophagic protein, Beclin1, by HIV-1 infection represents an essential mechanism in controlling HIV replication and viral-induced inflammatory responses in microglial cells. Existing antiretroviral therapeutic approaches have been limited in their ability to cross the blood-brain barrier effectively and recognize and selectively eliminate persistent HIV-infected brain reservoirs. In the present study and for the first time, the bio-distribution and efficacy of noninvasive intranasal delivery of small interfering RNA (siRNA) against the Beclin1 gene using the cationic linear polyethylenimines (PEI) as a gene carrier was investigated in adult mouse brain. Fluorescein isothiocyanate (FITC)-labeled control siRNA delivered intranasally was found in the cytoplasm of neurons and glial cells of the prefrontal cortex at 4 and 24 hours post-delivery, with no major adverse immune reaction encountered. Intranasal delivery of the siRNA targeting Beclin1 significantly depleted the target protein expression levels in brain tissues with no evidence of toxicity. Binding of siRNA to PEI-polymer was characterized and confirmed by Raman spectroscopy. These results indicate that the intranasal drug delivery allows for the direct delivery of the PEI-siRNA nano-complex to the central nervous system, which could potentially offer an efficient means of gene silencing-mediated therapy in the HIV-infected brain.

## Introduction

In spite of a dramatic reduction in human immunodeficiency virus (HIV)-associated dementia (HAD), HIV RNA copy number and great improvement in the survival rate among people living with HIV using long-term combined anti-retroviral therapy (cART), virus persistence continues in latent virus reservoirs and the virus can re-populate once cART is discontinued [Reviewed in ref.^1^. The blood-brain barrier (BBB) tightly controls the trafficking of substances between the blood and brain and many of the widely used antiretroviral drugs available poorly penetrate the central nervous system (CNS). In addition, cART is not totally effective in controlling HIV replication in brain cells and does not directly target the inflammatory cascades which are believed to be the primary cause of neuronal injury or dysfunction related to HAD pathology^2–5^.

Despite their instability and potential to induce insertion mutagenesis, toxicity, and immunogenicity, in recent years the use of RNA interference (RNAi)-based therapeutics such as small interfering (si)/short hairpin (sh)RNAs to mediate silencing of gene expression has shown exciting prospects for the development of novel therapeutic strategies^6^. With brain being the target organ for HAD, it represents an additional impediment in delivery of si/shRNA due to the presence of the BBB^7,8^. Encapsulating siRNA within nanoparticles not only protects the RNA from nucleases but also shields its overall negative charge, which can prolong the *in vivo* circulation life in blood and facilitate its passage across the cell membrane to the cytoplasm of target cells^9^. As shown by others, numerous non-viral strategies for si/shRNA delivery based on lipids^10,11^, biocompatible polymers/dendrimers^12,13^, polypeptides^14^ and inorganic materials^15,16^ have been implemented and are currently under investigation. One in particular is the biodegradable linear polymer cationic polyethylenimines (PEI) which have been used efficiently for the delivery of plasmid DNA^17^ and siRNAs^18,19^. The success by which the nanoparticle-based RNAi is delivered to brain is dependent on several factors, including the route of administration^20^. The non-invasive intranasal method of drug delivery is not obstructed by the BBB and allows molecules that do not cross the BBB direct access to the CNS^21^. Delivery from the nose to the CNS occurs within minutes along both the olfactory and trigeminal neural pathways via an extracellular route and does not require drug to bind to any receptor or axonal transport^21^. Efficacy of therapeutics administered intranasally in mice has been demonstrated in a number of studies including for insulin in diabetic mouse models^22–24^ and deferoxamine in Alzheimer’s mouse models^25,26^.

In the present study, we investigated the therapeutic potential of intranasal siRNA delivery in adult mice using siRNA targeting the Beclin1 (*Atg6*) gene in complex with PEI. Beclin1 is an essential protein in regulating autophagic activity by acting as a platform to recruit and activate positive regulators such as phosphatidylinositol-3 kinase (PI3KCIII/Vps34) complexes that mark membranes for autophagosome generation and facilitate autophagosome fusion with lysosomes, and also recruit negative regulators, thereby blocking vesicle nucleation and autophagy maturation [reviewed in ref.^27^. We have discovered that activation of the host autophagic pathway by HIV-1 infection represents an essential mechanism in controlling viral replication and viral-induced inflammatory responses in microglial cells^28^. The purpose of this study was to examine the bio-distribution and efficiency of intranasal delivery of siRNA to the brain and to investigate target gene silencing efficiency and possible toxicity in different brain regions. Altogether, our data shows optimization of the efficacy of siBeclin1 in attenuating HIV replication and HIV-induced inflammatory responses *in vitro* for *in vivo* use, the feasibility of using PEI reagent as gene carrier, and the bio-distribution of the nano-complex in brain via intranasal delivery.

## Results

### Formation of PEI-FITCsiRNA nanoplexes used for intranasal administration

**Figure 1:**
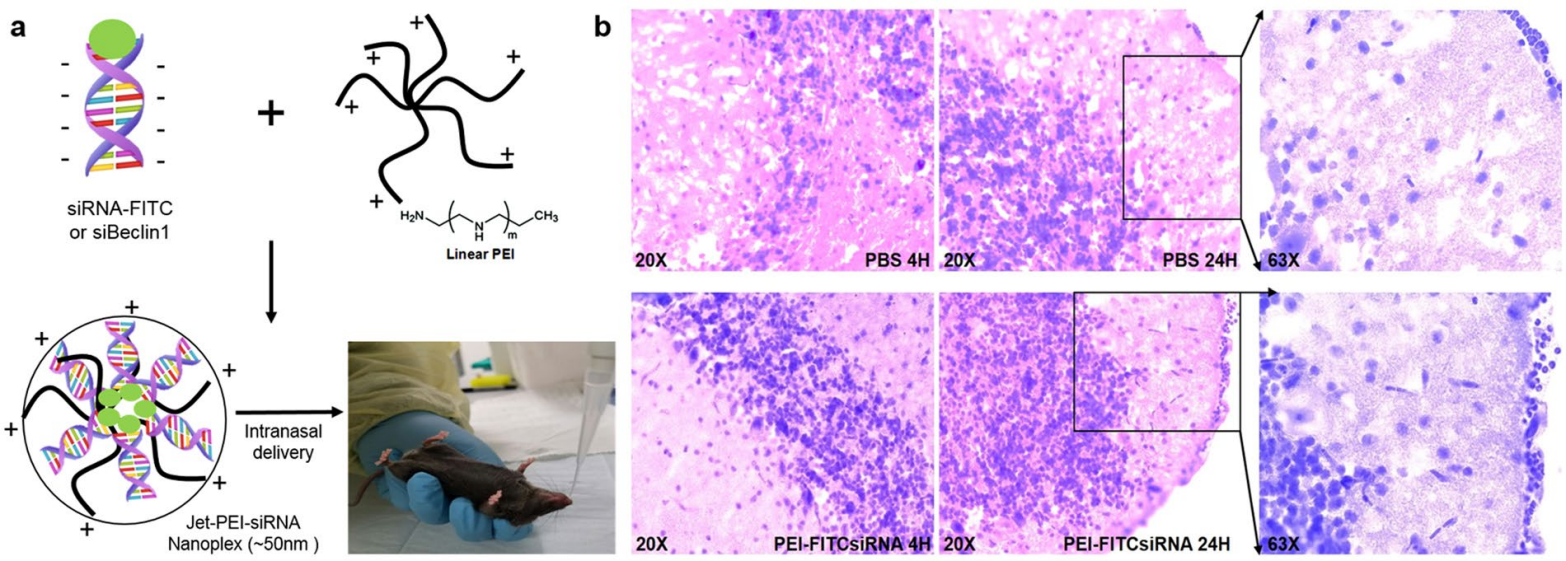
PEI-siRNA formulation and mouse brain histology after intranasal administration of nanoplexes. (**A**) Schematic representation of the PEI-siRNA formulation and intranasal administration to adult C57BL/6 J mice (n = 3/treatment). (**B**) Representative images of Hematoxylin-eosin stained sections of brain tissues after 4 hours (at 20X magnification) and 24 hours of intranasal administration of PBS (upper panel) and PEI-FITCsiRNA (lower panel) at 20X and 63X magnification.

Fluorescein isothiocyanate (FITC)-labeled control siRNA (2 µg) or Beclin1 siRNA (16 µg) forms a complex through binding with the linear polyethylenimine (PEI)-based cationic polymer, *in vivo* jetPEI, which can be used as gene carrier for intranasal delivery (Fig. 1A). *In vivo* jetPEI is a biodegradable polymer, which shows maximum siRNA delivery efficiency at an N/P ratio of 7.0^29–31^. H&E staining of prefrontal cortex from mouse brain tissues after 4 and 24 hours administration (Fig. 1B) showed normal cell morphology with minimal vacuolation in PEI-FITCsiRNA and PBS treated tissues. Moreover, the granule cell layer appeared undisrupted and there were no signs of aberrant morphology of purkinje neurons and astrocytes. This suggests that intranasal administration of PEI-FITCsiRNA nanoplexes does not exert any noticeable tissue toxicity.

### Cell-type distribution of PEI-FITCsiRNA nanoplexes after intranasal delivery in mouse brain

**Figure 2:**
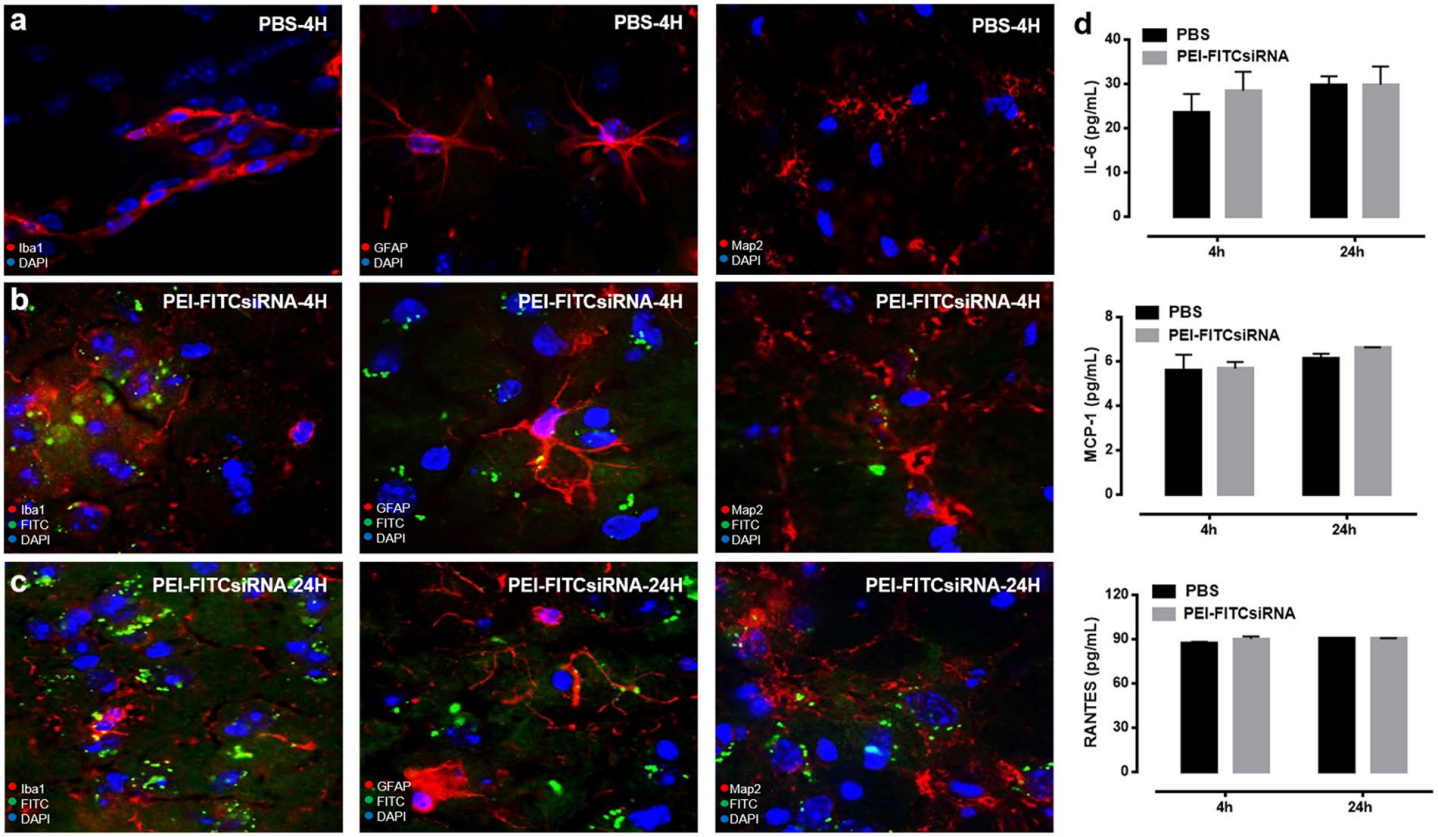
Cell-type distribution of PEI-FITCsiRNA in mouse brain following intranasal administration. Representative images of brain sections immunolabeled with antibodies to the microglial marker, Iba1 (left panel), astrocyte marker, GFAP (middle panel), and neuronal marker, Map2 (right panel), in *red* for (**A**) PBS treatment, and PEI-FITCsiRNA (indicated by *green* fluorescence) administration for (**B**) 4 hours and (**C**) 24 hours. DAPI (*blue)* staining indicates cell nuclei at 63X magnification. (**D**) The other half of brain tissues was used to detect the indicated cytokines and chemokines by ELISA after 4 and 24 hours. Values were determined from standard curves and are presented as the mean ± the standard error of the mean (S.E.M.) of three independent experiments.

We next examined the efficiency of intranasal siRNA delivery in normal mouse brain by triple fluorescence imaging using the FITC-labeled control siRNA, cell-type-specific immunolabeling, and DAPI staining (Fig. 2). Four hours post-delivery mouse brain (frontal cortex) treated with PBS was examined with DAPI (blue), the microglial marker, Iba1 (red), the astrocyte marker, GFAP (red), and the neuronal marker, Map2 (red), respectively (Fig. 2A). In similar fashion, mouse brains treated with PEI-FITCsiRNA at 4 hours post-delivery (Fig. 2B) were examined with antibody against microglia (left panel), astrocytes (middle panel) and neurons (right panel), DAPI, and green fluorescence. Figure 2C represents mouse brain (frontal cortex) treated with PEI-FITCsiRNA after 24 hours. The data shows the successful delivery of siRNA in different cell types of the prefrontal cortex as soon as 4 hours after intranasal administration, and the presence of siRNA was still detected after 24 hours of administration. The other half of the brain hemisphere was minced and used to detect for inflammatory responses looking at the release of IL-6, MCP-1 and RANTES by ELISA which showed no significant increase with PEI-FITCsiRNA when compared to PBS treated mice at 4 and 24 hours (Fig. 2D). Overall, this data suggests that PEI-FITCsiRNA nanoplexes can be successfully delivered to the brain and colocalized in brain cells without any significant inflammatory molecule release.

### Presence of PEI-FITCsiRNA nanoplexes in lungs after intranasal delivery

**Figure 3:**
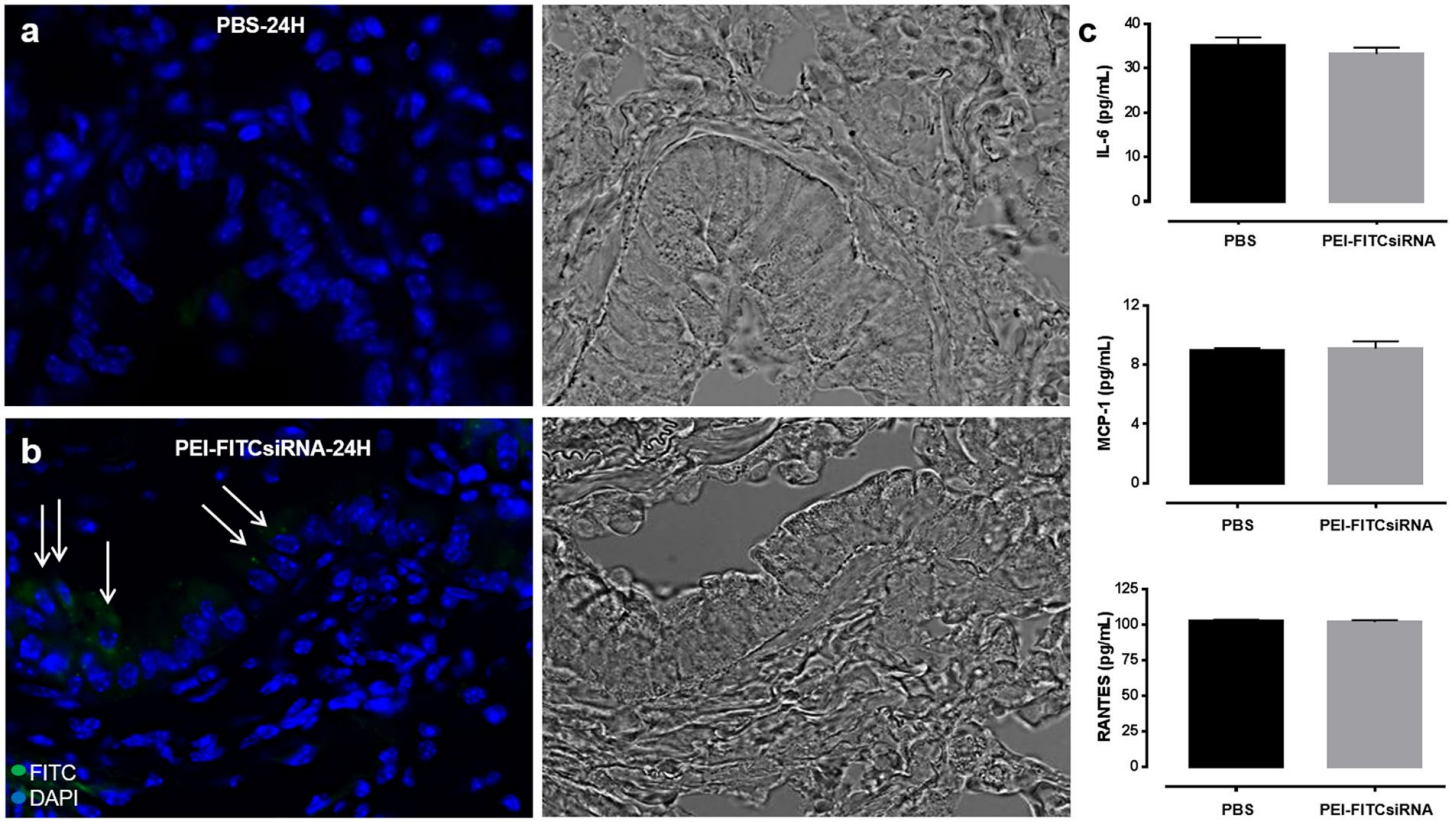
Distribution of PEI-FITCsiRNA nanoplexes in mouse lung following intranasal administration. Representative images of lung sections stained with DAPI (*blue*) to label cell nuclei after 24 hours of intranasal administration of PBS (A, left panel) and PEI-FITCsiRNA (B, left panel). Arrows indicate the presence of *green* fluorescent signals in the ciliated epithelial cells at 63X magnification. (A,B, right panel) Respective bright field images for morphology confirmation. (**C**) The other half of lung tissues was used to detect the indicated cytokines and chemokines by ELISA. Values were determined from standard curves and are presented as the mean ± the standard error of the mean (S.E.M.) of three independent experiments.

Since the lungs are directly connected with the nasal cavity through the pharynx and upper and lower respiratory passages^32^, we determined whether off-target organs, such as the lungs, were affected by intranasal administration at 24 hours post-delivery. The distribution of the nanoplex as measured by green fluorescent signals were found in ciliated epithelial cells (arrows) when compared to PBS treated lungs (Fig. 3A,B, left panel). In addition, based on brightfield microscopy, no morphological differences were detected in the lung tissues with the different treatments (Fig. 3A,B, right panel). Since we were able to detect green fluorescence, we measured inflammation by ELISA using the other half of the lung. Release of IL-6, MCP-1 and RANTES was measured and showed no significant increase in pro-inflammatory responses with PEI-FITCsiRNA when compared to PBS treated mice (Fig. 3C). Overall, the data confirms the effective delivery of PEI-FITCsiRNA nano-formulation to the brain with minimal off-target effects to the lungs.

### Titration of siBeclin1 in HIV-infected primary human microglia

**Figure 4:**
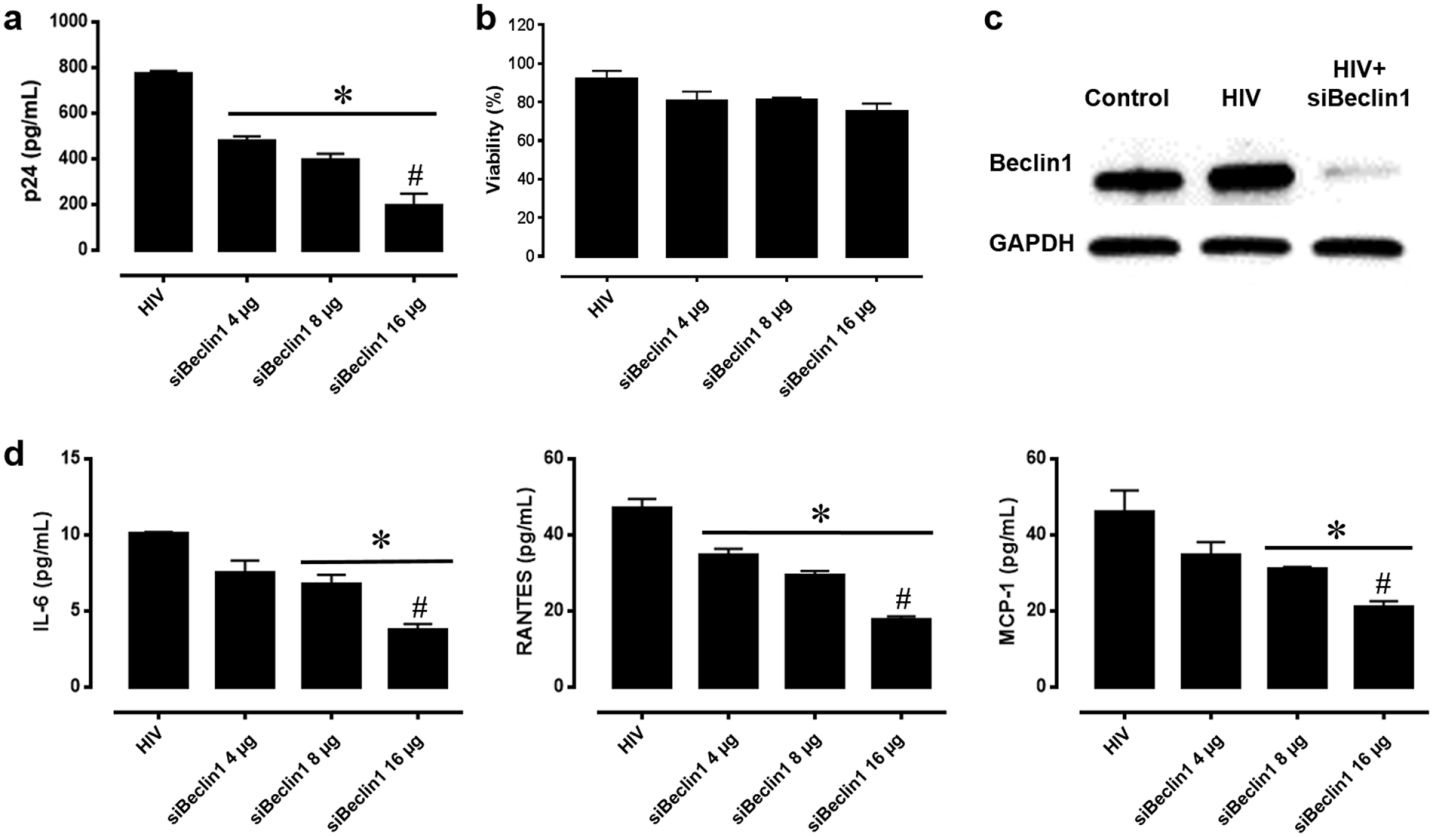
Titration of siBeclin1 achieved attenuation of HIV-1 replication and -induced inflammation *in vitro*. (**A**) HIV-1 replication in human microglia transfected with the indicated amounts of siBeclin1 was measured using HIV-1 p24^*gag*^ protein ELISA. Values were determined from standard curves and are presented as the mean ± the standard error of the mean (S.E.M.) of three independent experiments (^*^p < 0.05 vs. HIV; ^#^ vs. siBeclin1 4 µg). (**B**) Cell viability assay performed following the indicated treatments showed no significant microglial cellular toxicity. Data are presented as percentages of viable cells ± the S.E.M. from three independent experiments. (**C**) Cell lysates from microglia with the indicated treatments were subjected to immunoblotting with antibodies to Beclin1 and GAPDH as a loading control. (**D**) Corresponding cell culture supernatants used for p24 ELISA were used to detect the cytokines and chemokines IL-6, RANTES and MCP-1 by ELISA. Values were determined from standard curves and are presented as the mean ± the S.E.M. of three independent experiments (^*^p < 0.05 vs. HIV; ^#^ vs. siBeclin1 4 µg).

In our recently published data we confirmed that 2 µg of siBeclin1 was efficient in attenuating HIV replication and HIV-induced inflammation using *in vitro* cell culture conditions^16,28^. However, since we detected presence of FITCsiRNA in the lungs after intranasal administration (Fig. 3), we performed a titration curve using increased amount of siBeclin1 (4, 8 and 16 µg) in order to ensure that sufficient Beclin1 siRNA would be delivered to the brain in animal studies. As expected, viral titer as detected by HIV-1 p24^*gag*^ protein ELISA showed a significant reduction in the supernatant of microglia after 24 hours transfection with 4 µg siRNA, although viral reduction was more efficient at 16 µg (Fig. 4A). In fact, viral titer was reduced by ~50% at 4 µg, and by ~75% at 16 µg treatment when compared to HIV-1-infected cells alone. To determine whether the functional effects of siBeclin1 at the different amounts can lead to cell death, viability was assessed after 24 hours treatment using a live/dead cell assay. No significant differences were observed in the viability of infected microglial cells under the different treatment conditions (Fig. 4B). The silencing efficiency of siBeclin1 (at 16 µg) in HIV-1-infected microglia, as determined by Western blotting analysis, showed a robust inhibition of Beclin1 protein expression after 24 hours transfection (Fig. 4C). Inflammatory responses in the supernatant of microglial cells measured by ELISA showed significant reductions in the levels of IL-6, RANTES and MCP-1, and the reduction was dependent on the siBeclin1 concentration used (Fig. 4D). Twenty-four hours post-treatment with 4, 8 and 16 µg of siBeclin1 caused a significant decrease of 25%, 35%, and 55% in the release of IL-6, respectively, a significant decrease of 26%, 30% and 54%, respectively, in the release of RANTES and a significant decrease of 27%, 32% and 50%, respectively, in the release of MCP-1 when compared to HIV-infected microglial cells alone. Efficacy of siBeclin1 was also measured in HIV-infected human astrocytes and showed similar pattern in viral attenuation and in the reduction of inflammatory molecules as with microglia cells (data not shown). In summary, the *in vitro* data suggests that siBeclin1 (at 16 µg) was most effective in attenuating HIV titer and viral-induced inflammation in glial cells and therefore chosen in subsequent *in vivo* intranasal experiments.

### Raman spectroscopic evaluation of the PEI-siBeclin1 formulation for intranasal administration

**Figure 5:**
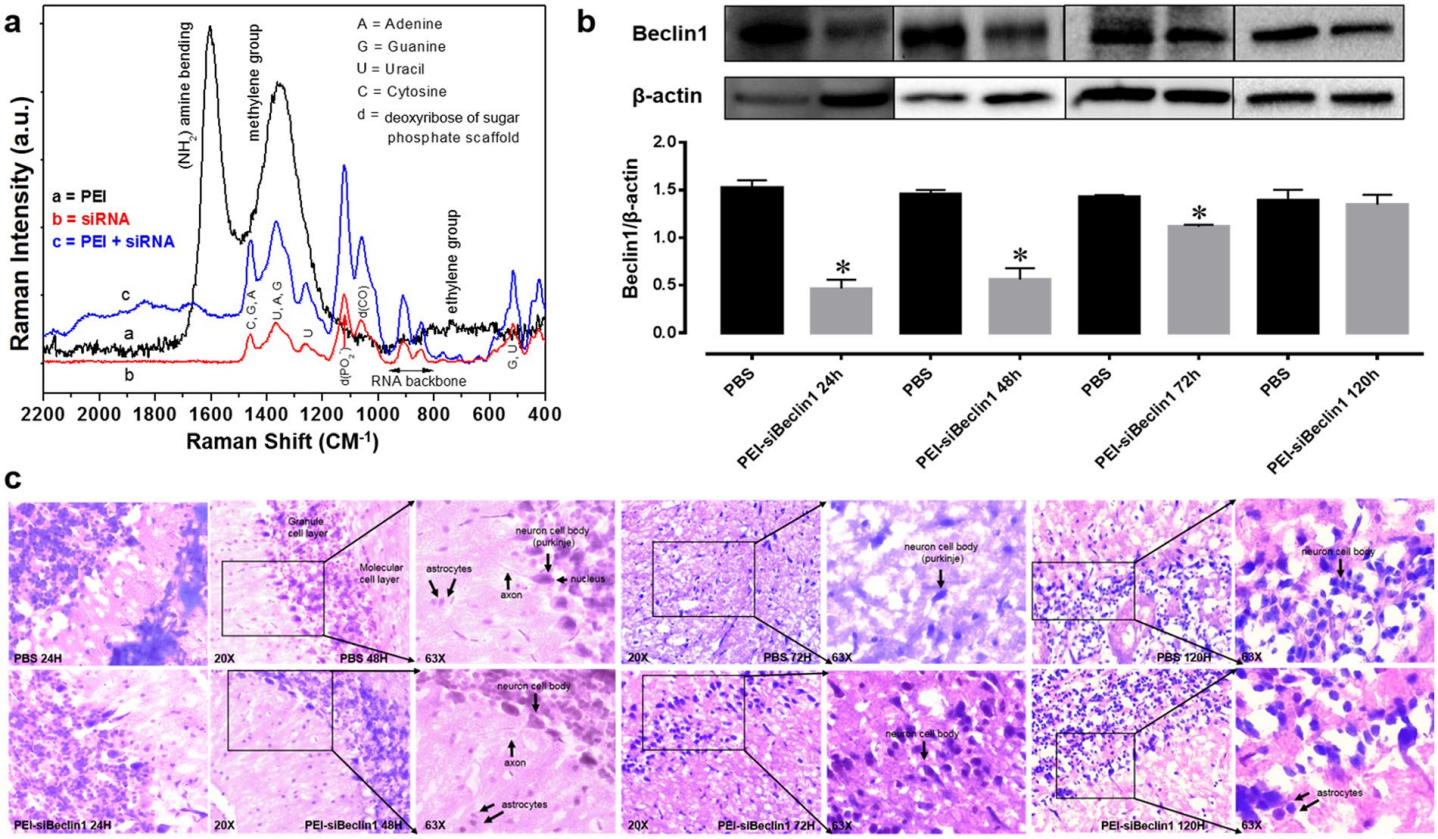
PEI-siBeclin1 binding analysis and functionality assessment following intranasal administration. (**A**) Raman spectroscopic study of PEI (curve a), Beclin1 siRNA (curve b), and the formulation consisting of PEI and siBeclin1 bound as a complex (curve c). Abbreviations used are defined as indicated. (**B**) Beclin1 silencing efficiency was measured by western blotting analysis using lysates from brain tissue after intranasal administration of the indicated treatments. Representative western blot images and graphical representations showing Beclin1 expression normalized to β-actin (loading control) at the indicated time points are presented. Error bars show the standard error of the mean (S.E.M.) of three independent experiments (^*^p < 0.05 vs. PBS). (**C**) Representative images of Hematoxylin-eosin stained sections of brain tissues at the indicated time points after intranasal administration of PBS (upper panel) and PEI-siBeclin1 (lower panel) at the indicated magnifications. Arrows and labels indicate distinctive cellular morphology.

Prior to intranasal administration, we assessed the binding efficiency of PEI-siBeclin1. The assessment of functional groups exhibited by the PEI-siBeclin1 formulation was characterized stepwise using Raman spectroscopy as shown in Fig. 5A. The Raman spectroscopic features were characterized in the region ranging from 2200 to 400 cm^−1^ because PEI and base pairs along with major functional groups of nucleic acids are more responsive in the selected region^33^. The Raman spectrum of PEI (curve a) exhibited an intense sharp band at 1600 cm^−1^ that is due to the amine group (N-H bending) and a broad intense band at 1380 cm^−1^ attributed to the vibration of C-N and C-H bending in the methyl group^33,34^. A low intensity weak band observed at 735 cm^−1^ is attributed to rocking and vibration of ethylene of PEI^33^. The Raman spectrum of siBeclin1 exhibited all optically active modes which corresponds to bases present in nucleic acid (curve b). The observed Raman bands at 510 cm^−1^ (G, U), 580 cm^−1^ (C;G), 630 cm^−1^ (A), 682 cm^−1^ (G), 760 cm^−1^ (U;d), 1050 cm^−1^ (d-CO), 1121 cm^−1^ (d-PO_2_^−^), 1253 cm^−1^ (C), 1365 cm^−1^ (U;A;G), and 1450 cm^−1^ (d (CH_2_)) are present in the nucleic acid chain^33,35^. The spectral region ranging from 800 to 1000 cm^−1^, which corresponds to the RNA backbone, exhibited Raman bands at 840 and 910 cm^−1^ that are attributed to O-P-O stretching vibration related with DNA structure and the phosphate-ribose backbone, respectively^33–35^.

Next, we observed that the PEI-siBeclin1 formulation exhibited all the Raman responsive modes as shown by PEI and siBeclin1 alone, suggesting the presence of Beclin1 siRNA and PEI. However, the intensity of the very sharp intense band at 1600 cm^−1^ exhibited by PEI was reduced remarkably and shifted to a higher wavelength, ~1700 cm^−1^. This might be due to possible binding between the amine group of PEI and base pairs of siBeclin1. Also, the other Raman bands exhibited by the PEI-siBeclin1 formulation were broader than those observed for siBeclin1. This may be due to possible ionic interaction between PEI and siBeclin1. The results of the Raman study showed that PEI and Beclin1 siRNA exhibited bands related with the functional groups of the individual materials. However, the Raman bands exhibited by the formulation correlated with functional groups of PEI and siBeclin1, confirming complex formation without losing functionality, as discussed and validated through *in vitro* assays.

### Intranasal delivery and efficacy of PEI-siBeclin1 nanoplexes in mice

Next, we assessed the bio-distribution and efficacy of siBeclin1 (16 µg) delivered intranasally in the brain of normal adult mice (Fig. 5). We examined whether intranasally delivered siBeclin1 caused target gene silencing in brain regions of the prefrontal cortex (Fig. 5B) and striatum (data not shown). The striatum and prefrontal cortex are areas of interest, since high levels of HIV are found in the striatal region of post-mortem patients and HIV causes striatal dysfunction leading to motor deficits^36–38^. Twenty-four, 48, 72 and 120 hours post-delivery of the nanoplex, Beclin1 silencing in the frontal cortex by intranasally delivered siBeclin1 was confirmed by immunoblot analysis. Protein expression was significantly downregulated by 65% after 24 hours and 43% after 48 hours (Fig. 5B), while downregulation by siBeclin1 was minimally detected after 72 hours and Beclin1 expression was recovered after 120 hours (Fig. 5B). Release of IL-6, MCP-1 and RANTES by ELISA showed no significant increase with PEI-siBeclin1 when compared to PBS treated mice (data not shown). H&E staining from the prefrontal cortex of PEI-siBeclin1 treated brain tissues at 24, 48, 72 and 120 hours post-delivery did not show myelin vacuolation, a characteristic indication of neurotoxicity, when compared with the PBS control group (Fig. 5C). There was also no noticeable neuronal swelling detected, and the normal presence of astrocytes in pairs was observed in tissues exposed to PEI-siBeclin1 and PBS (arrows). Similarly to the brain, PEI-siBeclin1 did not cause detectable tissue damage in the lung, liver, kidney, spleen or heart after intranasal delivery (see Supplementary Fig. S1). Overall, the data shows efficient delivery of siBeclin1 and downregulation of Beclin1 protein expression using *in vivo* jetPEI, and most importantly that intranasal delivery of siBeclin1 did not cause noticeable detrimental effects to the brain and lung tissues as well as other main organs.

## Discussion

In recent years, the efforts made to develop RNAi-based therapies to mediate silencing of gene expression have led to productive research in the field of numerous infectious diseases affecting humans, including HIV^39–45^. Despite the high potential use of antisense therapy, its clinical application is limited mainly due to its short half-life *in vivo*, lack of target cell specificity, and poor transport across the cell membrane^46^. However, packaging siRNA within nanoparticles has proven to protect nucleotides from the complex biological environment by shielding the overall negative charge of siRNA, increasing its stability and preventing RNase cleavage. Various strategies have been examined to improve delivery efficiencies of drugs to the brain. The application of drugs encapsulated into non-viral vectors, like synthetic nanoparticles, was found to enhance delivery to neuronal cells and the brain itself ^21,26,47^. Using electro-magnetic nano-material (CoFe2O4@ BaTiO3; MENP) bound to siRNA targeting Beclin1, we showed that MENP-siBeclin1 can cross an artificial BBB and attenuate the neurotoxic effects of HIV-1-infected microglia following on-demand release of siRNA using an *in vitro* primary human BBB model^16^.

The present study demonstrates the successful intranasal delivery of PEI-siRNA nanoplexes and subsequent silencing of Beclin1 protein expression in different brain regions including the cerebral cortex and striatum. Targeting Beclin1 is of relevance since numerous evidence has shown that autophagy is a critical target for HIV during the viral life cycle which has led to an increasing effort to understand the role of autophagy in those cells affected by HIV infection^48–50^. The autophagy pathway engulfs and degrades cytoplasmic cargo through lysosomal degradation [reviewed in ref.^27^, and plays a crucial role in defense against microbial infections, neurodegenerative disorders, cancer and aging^51^. Specific autophagy-inducing agents are being considered for therapeutic treatment and prevention of a broad range of human diseases^52–55^. In Fig. 1, we developed a nanoplex composed of linear cationic polyethylenimines (PEI) bound to siRNA that was delivered through intranasal administration in adult mice. PEI has a high transfection efficiency both *in vivo* and *in vitro*, and was initially used as an efficient non-viral transfection reagent for the delivery of plasmid DNA^17^. In recent years, PEI-mediated delivery of nucleic acids was extended towards siRNAs as well^18,19^. Due to its high cationic charge density and a large number of protonable nitrogen atoms, PEI is able to form stable, water-soluble, non-covalent complexes with nucleic acids. According to the literature, these PEI complexes are efficiently taken up by cells through endocytosis and, based on their unique property of acting as a “proton sponge”, the continuous proton influx induces endosome osmotic swelling and rupture which provides an escape mechanism for siRNA particles intracellularly without the support of endosomal disruptive agents for lysosomal escape^56^. The proton sponge effect buffers the endosomal pH and protects the siRNA from degradation^56^. It is important to note that the transfection reagent used here, *in vivo*-jetPEI (Polyplus), is being used in preclinical studies and phase I and II clinical trials, although mainly for cancer therapies^57,58^. As shown in Fig. 2, PEI-siRNA nanoplexes can be efficiently delivered to different cell types in the brain via the intranasal route, without noticeable evidence of toxicity (Fig. 1B), although low levels of green fluorescence were detected in the lungs from animals treated with PEI-FITCsiRNA after 24 hours (Fig. 3B). We have previously shown that silencing Beclin1 with siBeclin1 (2 µg) represents an essential mechanism in controlling HIV replication and viral-induced inflammatory responses in microglial cells *in vitro*^28^. Low level detection of siRNAFITC in the lungs (Fig. 3) prompted us to perform a titration curve to determine a higher, yet effective concentration of siBeclin1 in attenuating HIV replication and viral-induced inflammation in human glial cells for *in vivo* intranasal delivery use (Fig. 4). Binding and PEI-siBeclin1 nanoplex formation as determined by Raman analysis (Fig. 5) and a robust downregulation of the Beclin1 protein as determined by western blotting (Fig. 5), without exerting noticeable toxicity to the brain tissue, provides a proof-of-concept on the functionality of our designed nanoplex and intranasal delivery. Our study used intranasal delivery, which is a noninvasive method to rapidly deliver drugs directly from the nasal mucosa to the brain and spinal cord bypassing the BBB, with the aim of treating CNS disorders while minimizing systemic exposure^21^. Although numerous studies have demonstrated the efficacy of drug delivery via the intranasal route to treat animal models of CNS diseases^59–62^, importantly, this mode of transmission has also shown great success in drug delivery in several human studies^63–66^, including clinical phase trials^67–69^. In one clinical trial, human subjects receiving intranasal administration of insulin showed significant alterations in brain functions as well as improved memory and mood^70,71^, without affecting blood glucose and serum insulin levels. In another study, intranasal delivery of insulin in men lead to a decrease in food intake, enhancement of postprandial thermogenesis, and a decrease in postprandial serum insulin^72,73^. Furthermore, intranasal delivery of insulin in patients with amnesic mild cognitive impairment resulted in modulated plasma levels of amyloid beta and improved memory, attention, and functional status, suggesting a possible role for intranasal delivery of insulin in the treatment of Alzheimer’s disease^66^. Intranasal administration of oxytocin, a neuropeptide with a wide range of effects on human behavior, has been shown to increase social interactions, reduce anxiety and psychological stress, and improve emotional recognition in youths with autism spectrum disorders^74,75^. Exogenous administration of the antioxidant, Glutathione, to patients with Parkinson’s disease (PD) via intranasal delivery was proven safe and effective with anecdotal case reports of improvement^68^. Since changes in Glutathione have been reported in numerous other disorders of the CNS, including schizophrenia, dementia, Huntington’s disease, and autism, the intranasal therapeutic potential of Glutathione may not be limited to patients with PD^68^. Our *in vivo* mouse model of intranasal administration of siRNA is greatly relevant to ongoing studies exploring the use of intranasal delivery of drugs for targeting CNS disorders and it is particularly novel as a therapeutic strategy for HIV neurological complications.

Despite the novelty of this approach, the major challenge with intranasal delivery is to achieve an optimum amount of the nanoplexes delivered to the CNS and protection of the lungs from exposure. Therefore, formulational modifications and possible systemic side effects of intranasal delivery require future study. Ongoing studies in our laboratory are assessing whether liver and kidney functions are affected after intranasal administration of siBeclin1, in conjunction with histopathology and blood toxicity profiles. In conclusion, the present study shows the usefulness of linear PEI as a gene carrier *in vivo* and the effectiveness of intranasal delivery of Beclin1 siRNA as a promising therapeutic tool for attenuating HIV-1 and viral-induced inflammation in the brain. Future applications in our laboratory will be analyzing the effectiveness of PEI-siBeclin1 in attenuating HIV using viral-infected humanized severe combined immunodeficiency (SCID) mice.

## Materials and Mehods

### Cell culture

Commercially obtained primary human microglia (ScienCell Research Laboratories, Carlsbad, CA, USA; catalog # 1900) were cultured as per the manufacturer’s protocol.

### *In vitro* HIV-1 infection

Human microglia were infected with HIV-1_SF162_ (p24 = 1 ng/mL; from Dr. Jay Levy^76^ obtained through the NIH AIDS Research and Reference Reagent Program (Germantown, MD, USA) as performed previously^28,77,78^. HIV-1 infection was confirmed by quantification of p24 levels in culture supernatants using the RETRO-TEK HIV-1 p24 antigen ELISA kit (ZeptoMetrix, Buffalo, NY, USA) as performed previously^28,77,78^.

### Transfection of Beclin1 siRNA into microglial cells

HIV-infected human microglia were transfected with Beclin1 siRNA purchased from Santa Cruz Biotechnology (Santa Cruz Biotechnology, Santa Cruz, CA, USA; catalog # sc-29797) using INTERFERin-PEI reagent (Polyplus-transfection, New York, NY, USA) for 24 and 48 hours as per the manufacturer’s protocol.

### Intranasal administration of siRNA-PEI nano-complexes into C57BL/6 J mice

C57BL/6 J mice (stock # 000664) were procured from The Jackson Laboratory (Bar Harbor, ME, USA) and bred in the animal facility at Florida International University. All animal experiments were carried out in accordance with the approved IACUC protocol issued by Florida International University. In addition, the intranasal administration of siRNA was approved by the IACUC at Florida International University. Fluorescein isothiocyanate (FITC)-labeled control siRNA (Santa Cruz Biotechnology; catalog # sc36869), Beclin1 siRNA (Santa Cruz Biotechnology) or phosphate-buffered saline (PBS) was administered intranasally in normal adult mice using *in vivo* jetPEI reagent (Polyplus-transfection) according to the manufacturer’s protocol. Briefly, jetPEI and siRNA were diluted separately in a 10% glucose solution. The solutions containing siRNA and PEI nanoplexes were mixed and incubated for 15 min at room temperature. For titration calculation, the formulation corresponds to a nitrogen and phosphate (N/P) ratio of 7. Intranasal administration was performed on lightly anesthetized mice. Each mouse was placed on a sterile surgical pad and lightly stretched out to better hold the scruff. With a firm grip on the scruff, the mouse was turned on its back while still allowing the mouse to breathe and be comfortable. With the neck and chin flat and parallel to the pad, the tip of the pipettor containing the sample was placed near the left nostril of the mouse at a 45 degree angle, and about 5 µL of sample was administered to the nostril with a 2–3 sec interval in between for a total of 10 µL/nostril. The mouse was held in this position for 5 sec or until it regained consciousness, then the administration step was repeated for the other nostril for a total of 20 µL/mouse. After the mouse had received all drops, the animal was kept restrained on its back until the material disappeared into the nares and then returned back to its cage. After 4, 24, 48, 72 and 120 hours mice were sacrificed and half of the brain hemisphere, liver, lungs, heart, spleen and kidneys were cryopreserved by serial exposure to 10 and 20% sucrose, embedded in Tissue Tek optimal cutting temperature compound (Sakura Finetek, Torrance, CA, USA) and used for histology and the other half was used for biochemical analysis as described below.

### Raman spectroscopic analysis

Raman spectrum of PEI, Beclin1 siRNA, and the PEI-Beclin1 siRNA formulation was recorded using a Nomadic Raman microscope from BaySpec (532 nm laser) (San Jose, CA, USA).

### Hematoxylin and eosin (H&E) staining

Brain, liver, kidney, lung, spleen and heart sections of 5 micron thickness were stained with H&E. Briefly, tissues were exposed to xylene and re-hydrated with absolute ethanol, 95 and 70% ethanol, followed by staining with hematoxylin dye for 15 min, prior to washing with distilled water. Subsequently, tissue sections were stained with eosin for 20 sec, dehydrated with gradient ethanol after washing with tap water, and tissues were then cleared by xylene and mounted using mounting media for visualization. Images were acquired using a Zeiss (Germany) inverted fluorescence microscope with a 560 Axiovision camera using 20, 40 or 63X objectives.

### Immunochemistry

Brain sections of 5 micron thickness were fixed in 4% paraformaldehyde, permeabilized with 0.1% Triton X-100, blocked in 10% milk/0.1% goat serum, and immunolabeled with anti-GFAP antibody (Millipore, Bedford, MA, USA; catalog # ab5804) at a 1:1000 dilution, anti-Iba1 (Santa Cruz Biotechnology, Santa Cruz, CA, USA; catalog # sc32725) at a 1:50 dilution and anti-MAP2 (Millipore, Bedford, MA, USA; catalog # MAB378) at a 1:100 dilution. Immunoreactivity was visualized with secondary antibodies from Molecular Probes (Carlsbad, CA, USA). DAPI staining was used to label cell nuclei. Images were acquired using a Zeiss (Germany) inverted fluorescence microscope with a 560 Axiovision camera.

### Western blotting

Whole cell lysates or brain tissues were prepared in RIPA buffer supplemented with a mixture of protease and phosphatase inhibitors and separated by SDS-PAGE for immunoblotting. Primary antibodies used were anti-Beclin1 (1:500) from Novus Biologicals (Littleton, CO, USA), anti-β-actin (1:200) from Santa Cruz Biotechnology and anti-GAPDH (1:1000) from Sigma-Aldrich (St. Louis, MO, USA). Primary antibodies were followed by incubation with a secondary antibody conjugated to horseradish peroxidase (Millipore, Billerica, MA, USA) used at a 1:1000 dilution. The immunoblots were exposed to SuperSignal West Femto Substrate (Thermo Scientific, Waltham, MA, USA) and visualized using a ChemiDoc imaging system (Bio-Rad, Hercules, CA, USA).

### ELISA

The other half of the brain and lung tissues were minced in RIPA lysis buffer and used to measure the levels of interleukin (IL)-6, monocyte chemotactic protein-1 (MCP-1), and regulated on activation, normal T cell expressed and secreted (RANTES) by ELISA (R&D Systems, Minneapolis, MN, USA) according to the manufacturer’s instructions. The optical density (O.D.) was read at A450 on a Synergy HTX plate reader (BioTek, Winooski, VT, USA).

### Viability assay

Viability of microglia was assessed using a live/dead cell fluorescence assay which combines fluorescent reagents to yield two-color discrimination of the population of live cells indicated by green fluorescence from the dead-cell population indicated by red fluorescence (ScienCell Research Laboratories). Cells were imaged using an inverted fluorescence microscope (Zeiss) and viable cells were manually quantified and reported as percent of viability.

### Statistical analysis

Data were analyzed using analysis of variance (ANOVA) techniques followed by Bonferonni’s post hoc test for multiple comparisons (GraphPad Prism 6 software, La Jolla, CA, USA). A value of *p* < 0.05 was considered significant.

## Electronic supplementary material

Supplementary Information
