# Author Correction: Intranasal drug delivery of small interfering RNA targeting Beclin1 encapsulated with polyethylenimine (PEI) in mouse brain to achieve HIV attenuation

**DOI:** 10.1038/s41598-018-23078-y

**Published:** 2018-03-14

**Authors:** Myosotys Rodriguez, Jessica Lapierre, Chet Raj Ojha, Ajeet Kaushik, Elena Batrakova, Fatah Kashanchi, Seth M. Dever, Madhavan Nair, Nazira El-Hage

**Affiliations:** 1 Department of Immunology, Florida International University, Herbert Wertheim College of Medicine, Miami, FL 33199 USA; 2 University of North Carolina, Eshelman School of Pharmacy, Chapel Hill, NC 27599 USA; 3 Laboratory of Molecular Virology, School of Systems Biology, George Mason University, Manassas, VA 20110 USA

Correction to: *Scientific Reports* 10.1038/s41598-017-01819-9, published online 12 May 2017

This Article contains typographical errors in the Acknowledgements section.

“We gratefully acknowledge the support of the National Institutes of Health (NIH)-National Institute on Drug Abuse (NIDA) grants R01 DA036154; R21 DA041287 to NEH and DA041749 to MN and National Institutes of Health (NIH)-National Institute on Mental Health (NIMH) R01 MH110262 to FK.”

should read:

“We gratefully acknowledge the support of the National Institutes of Health (NIH)-National Institute on Drug Abuse (NIDA) grants R01 DA036154; R21 DA041287 to NEH and DA034547 to MN and National Institutes of Health (NIH)-National Institute on Mental Health (NIMH) R01 MH110262 to FK.”

