# Supplementary material for: Intranasal drug delivery of small interfering RNA targeting Beclin1 encapsulated with polyethylenimine (PEI) in mouse brain to achieve HIV attenuation
Source: Sci Rep. 2017 May 12;7:1862. doi: 10.1038/s41598-017-01819-9 (PMC5431946; doi:10.1038/s41598-017-01819-9)

Intranasal drug delivery of small interfering RNA targeting Beclin1 encapsulated with polyethylenimine (PEI) in mouse brain to achieve HIV attenuation

Myosotys Rodriguez[1], Jessica Lapierre[1], Chet Raj Ohja[1], Ajeet Kaushik [1], Elena Batrakova[2], Fatah Kashanchi[3], Seth M. Dever[1], Madhavan Nair [1], Nazira El-Hage[1]*

1. Department of Immunology, Florida International University, Herbert Wertheim College of Medicine, Miami, FL 33199, USA.
2. University of North Carolina, Eshelman School of Pharmacy, Chapel Hill, NC 27599, USA.
3. Laboratory of Molecular Virology, School of Systems Biology, George Mason University, Manassas, VA 20110, USA.

Supplementary Fig. S1

Morphological analysis of different organs after intranasal administration of PEI-siBeclin1 nanoplexes

(A) Representative images of Hematoxylin-eosin stained sections of lung tissues after the indicated time points of intranasal administration of PBS (upper panel) and PEI-siBeclin1 (lower panel) at the indicated magnifications. (B) Representative images of Hematoxylin-eosin stained sections of heart, kidney, spleen and liver after the indicated time points of intranasal administration of PBS and PEI-siBeclin1 at 40X magnification.

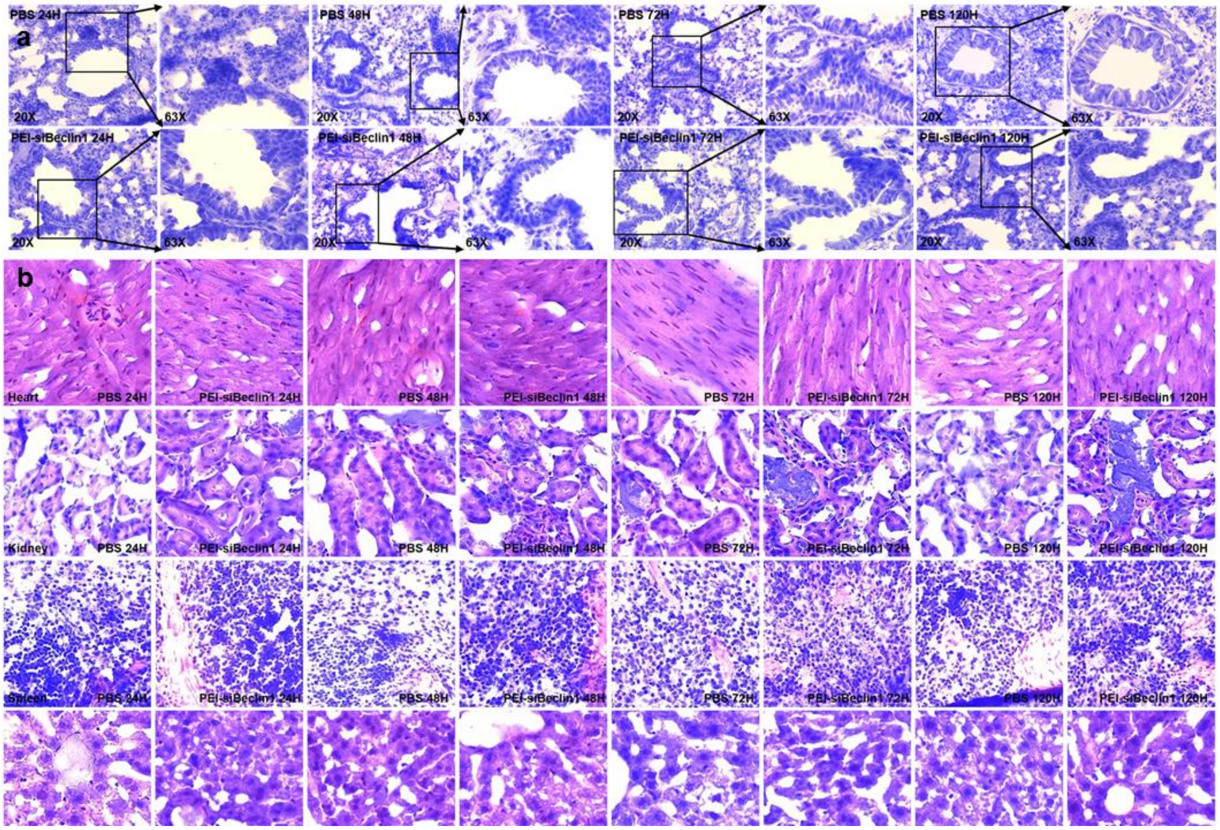

Supplement: Supplementary file 1 — Supplementary Information [file 41598_2017_1819_MOESM1_ESM.pdf]
